# Effects of Coupling Agent and Thermoplastic on the Interfacial Bond Strength and the Mechanical Properties of Oriented Wood Strand–Thermoplastic Composites

**DOI:** 10.3390/polym13234260

**Published:** 2021-12-05

**Authors:** Ziling Shen, Zhi Ye, Kailin Li, Chusheng Qi

**Affiliations:** MOE Key Laboratory of Wood Material Science and Utilization, Beijing Forestry University, Beijing 100083, China; shenziling@bjfu.edu.cn (Z.S.); ye.zhi037@gmail.com (Z.Y.); likailin2019@gmail.com (K.L.)

**Keywords:** wood–plastic composites, coupling agent, interfacial bonding, oriented strand–thermoplastic composites, wood veneer–thermoplastic composites

## Abstract

Wood–plastic composites (WPC) with good mechanical and physical properties are desirable products for manufacturers and customers, and interfacial bond strength is one of the most critical factors affecting WPC performance. To verify that a higher interfacial bond strength between wood and thermoplastics improves WPC performance, wood veneer–thermoplastic composites (VPC) and oriented strand–thermoplastic composites (OSPC) were fabricated using hot pressing. The effects of the coupling agent (KH550 or MDI) and the thermoplastic (LDPE, HDPE, PP, or PVC) on the interfacial bond strength of VPC, and the mechanical and physical properties of OSPC, were investigated. The results showed that coupling agents KH550 and MDI improved the interfacial bond strength between wood and thermoplastics under dry conditions. MDI was better than KH550 at improving the interfacial bond strength and the mechanical properties of OSPC. Better interfacial bonding between plastic and wood improved the OSPC performance. The OSPC fabricated using PVC film as the thermoplastic and MDI as the coupling agent displayed the highest mechanical properties, with a modulus of rupture of 91.9 MPa, a modulus of elasticity of 10.9 GPa, and a thickness swelling of 2.4%. PVC and MDI are recommended to fabricate WPCs with desirable performance for general applications.

## 1. Introduction

Wood–plastic composites (WPCs) are innovative wood-based composites manufactured using wood, thermoplastic, and additives through injection [1], extrusion [2], and hot-pressing molding processes [3,4]. Due to their excellent weatherability, dimensional stability, and mechanical properties, WPCs are widely applied in many fields [5]. The performance of a WPC is determined by the properties of its raw materials [6], wood content [7], and the interfacial bonding between the wood and the plastic [8].

Recent studies have demonstrated the effect of thermoplastic type on interfacial bonding [9]. Since wood is a porous material, molten plastic can penetrate its pores and form mechanical bonds. Plastics with a higher melt flow rate have better permeability, leading to tighter interfacial bonding between the wood and the plastic [10]. Bekhta et al. [11] concluded that samples prepared from low-density polyethylene (LDPE) showed the lowest bonding strength, and samples prepared using polyamide showed the highest bonding strength. Stadlmann et al. [12] reported tensile shear strengths of 8.7 MPa and 3.0 MPa for birch bonded with PA6 and polypropylene (PP), respectively. Gaugler et al. [13] investigated a new methodology for rapidly assessing interfacial bonding between fibers and thermoplastics, and analyzed the interfacial bonding between wood fibers and high-density polyethylene (HDPE), PP, polyurethane (TPU), and polylactic acid (PLA). The PLA composites display the highest shear strengths (8.0–9.0 MPa), far higher than the other three plastics. Cavdar et al. [14] observed that the composite prepared using smaller plastic polymer molecules had a higher tensile modulus. LDPE, high-density polyethylene (HDPE), PP, and polyvinyl chloride (PVC) are among the most used thermoplastics in the WPC industry. However, the interaction between thermoplastics and coupling agents is lacking, so the effects of the plastic type on the interfacial bond strength and mechanical properties of WPC with coupling agents require further investigation. 

Wood contains abundant hydroxyl and phenolic hydroxyl groups, making it highly popular; however, thermoplastics are nonpolar or weakly polar materials. The low compatibility between wood and plastic significantly impacts the interfacial bonding, leading to the low mechanical properties in the resulting composites [15]. Many studies have demonstrated that thermal modification [16,17], plasma treatment [18], and the addition of coupling agents [19,20] can improve interfacial bonding. 

Coupling agents are easy to handle and have lower energy consumption than thermal and plasma treatment methods used to improve the interfacial bonding between wood and thermoplastics. Furthermore, the chemical reactions between the coupling agent and raw materials enhance the compatibility and interfacial bonding between WPC components [21]. Liu et al. [22] demonstrated that silane coupling agents significantly improved the interfacial bonding between wood and HDPE, because the formation of Si-O-C bonds reduced the content of hydrophilic hydroxyl groups on the wood surface. Moreover, the A171-treated samples had a higher bonding strength than KH550-treated samples. Previous studies have demonstrated that methylenediphenyl-4,4′-diisocyanate (MDI) as a WPC coupling agent enhanced the mechanical properties due to the formation of stable urethane bonds between the isocyanate groups of MDI and the hydroxyl groups of wood [23,24]. Maleic anhydride is also an effective coupling agent for WPCs [25]. Despite these results, there is no unified conclusion about which combination of plastics and coupling agents has the best effect on the interfacial bonding in WPCs. Therefore, evaluating the effect of both coupling agent and plastic on interfacial bonding has great significance for preparing high-performance WPC. 

The interfacial bonding between wood and plastics is a key factor affecting the performance of WPC, including their mechanical strength, dimensional stability, and thermal properties. The samples with good bonding quality display a high bending strength, modulus of elasticity, and dimensional stability [11]. Preparing WPCs with thermoplastic films and veneers by hot pressing is an efficient method for interfacial evaluation [11,16]. However, it should be further verified whether this method is suitable for a WPC made of large strands using hot pressing.

This research aimed to establish an optimized combination of coupling agent and thermoplastic to produce oriented strand–thermoplastic composites (OSPCs) with desirable performances using hot pressing, and to verify that a higher interfacial bond strength between wood and thermoplastic improves the performance of an OSPC.

## 2. Materials and Methods

### 2.1. Materials

Poplar (*Populus tomentosa* Carr) veneers with dimensions of 400 × 400 × 1.2 mm^3^ and strands with lengths of 60–100 mm, widths of 15–20 mm, and thicknesses of 0.2–0.5 mm were obtained from Shandong province, China. All poplar veneers and strands were oven-dried until the final moisture content was below 3%. Thermoplastic films (HDPE, LDPE, PP, and PVC) with thicknesses of 0.1 mm were purchased from Wuhan Kaidi Plastic Products Company, and the plastic films were cut into 400 × 400 mm^2^ pieces. The coupling agent MDI (PM200), with 30.0–32.0% cyanate (–NCO) groups, was purchased from Yantai Wanhua Polyurethane Company. MDI was diluted with acetone at a mass ratio of 1:2 before use. KH550 was purchased from Guangzhou Yong Zheng Chemical Industry Co., Ltd., Guangzhou, China.

### 2.2. Methods

#### 2.2.1. Preparation of Veneer–Plastic Film Composites

**Figure 1:**
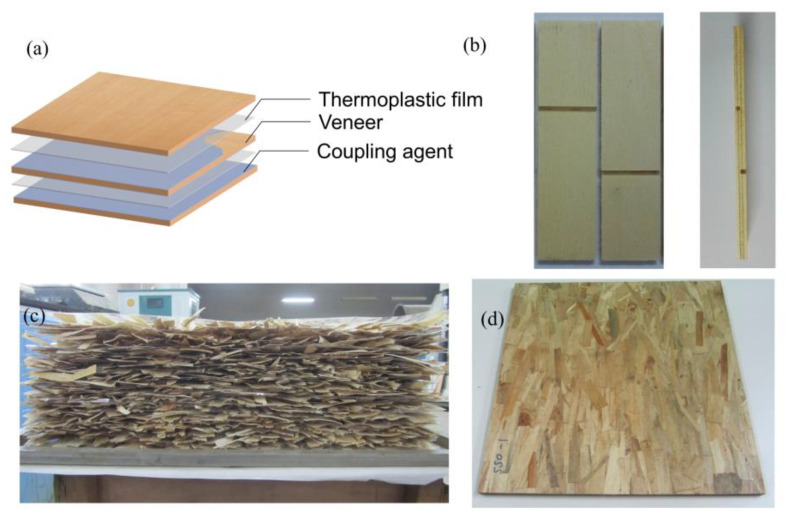
(**a**) Schematic diagram of the VPC; (**b**) interfacial bonding strength test samples; (**c**) forming mat of OSPC; (**d**) final OSPC product.

The structure of the veneer–plastic film composite (VPC) is shown in Figure 1a. Roughly 60.0 g/m^2^ coupling agent was coated onto the top surface of the first veneer, and one layer of plastic film was placed on the top surface of the veneer. Then, a second veneer, with both surfaces coated with a 60.0 g/m^2^ coupling agent and grain perpendicular to the bottom veneer, was placed on the first layer of the plastic film. Repeating the above steps, the third veneer was placed on the top, with its grain parallel to the grain of the first veneer.

The formed mat was hot-pressed under 1.0 MPa pressure at 180 °C for 6 min, and then cold-pressed for 20 min until the temperature dropped below 40 °C. The effects of coupling agent and thermoplastic type on interfacial bonding were investigated, and Table 1 shows the experimental design for fabricating the VPC and OSPC.

#### 2.2.2. Preparation of Oriented Strand–Thermoplastic Composites

The poplar strands were sprayed with 2.0 wt% coupling agent in a drum blender and then divided into 18 equal parts. A sheet of thermoplastic film was first placed on release paper. One portion of the strands was placed on the film with an orientation angle of ±20°, and the above steps were repeated until a sheet of thermoplastic film covered the surface (Figure 1c). The thermoplastic films accounted for 20.0 wt% of the final panel. The mat was hot-pressed at 180 °C, and its thickness was controlled using a maximum pressure of 5.0 MPa. After 10 min of hot pressing, the mat was rapidly transferred into a cold press and pressed for 20 min under 1.0 MPa until the temperature dropped below 40 °C. At least 15 mm was trimmed on each side to obtain a final board with a dimension of 400 × 400 × 12 mm^3^ and a target density of 0.8 g/cm^3^ (Figure 1d). 

### 2.3. Characterization

#### 2.3.1. Mechanical Strength

The VPC interfacial bond strength was evaluated according to GB/T 9846. The test samples (Figure 1b) were stretched at a cross-head speed of 10 mm/min using a universal material testing system. Thirty specimens were tested for each composite. The dry bond strength was measured after keeping the temperature and relative humidity under 20 °C and 65% for 7 days, until the weight was consistent. The wet bond strength was measured after submerging the samples in hot water (63 ± 3 °C) for 4 h.

The modulus of rupture (MOR) and modulus of elasticity (MOE) were evaluated according to ASTM D1037 using the three-point bending method. The cross-head speed was 5 mm/min during the static bending test, and 2 mm/min for the tensile test. Five specimens were tested for each sample.

#### 2.3.2. Dynamic Mechanical Analysis (DMA)

The dynamic mechanical properties were analyzed using a TA Instruments Q800 at a frequency of 1 Hz, an amplitude of 20 μm, and a temperature range from room temperature to 180 °C. The storage modulus (*E’*) and loss tangent (*tanδ*) of VPCs with different coupling agents and thermoplastics were evaluated.

#### 2.3.3. Morphological Examination

The morphology of the VPC was examined using an S3400 scanning electron microscope (SEM). SEM micrographs were used to investigate the interface between the wood and thermoplastics. The specimens were sputter-coated with gold for 1 min and dried in a vacuum at 100 °C for 1.5 h prior to the study.

#### 2.3.4. Dimensional Stability

The thickness swelling (TS) and water absorption (WA) were evaluated according to ASTM D1037, and all samples were soaked in distilled water for 24 h. Five specimens were tested for each sample.

## 3. Results and Discussion

### 3.1. Interfacial Bond Strength of VPC

**Figure 2:**
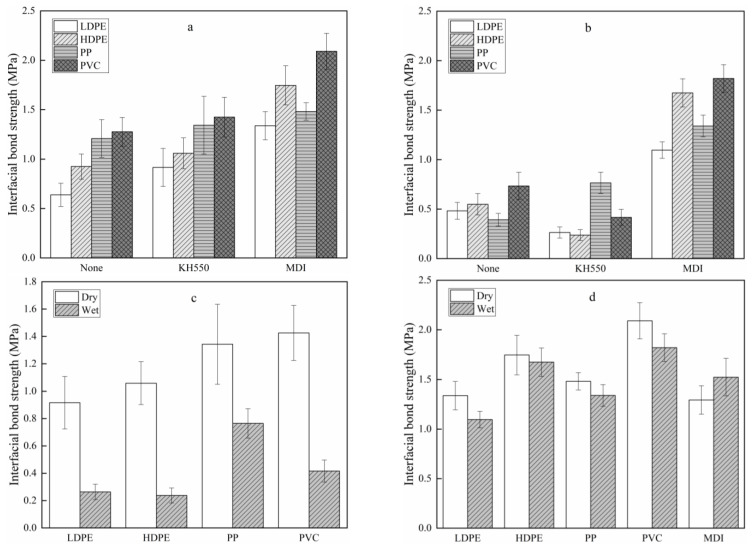
Interfacial bond strength of VPC under: (**a**) dry conditions; (**b**) wet conditions. The interfacial bond strength of VPC with the coupling agent: (**c**) KH550; (**d**) MDI.

The effects of coupling agent and thermoplastic type on the interfacial bond strength of VPCs are shown in Figure 2. The coupling agent and plastic type had an evident influence on the interfacial bond strength of the VPC. Under dry conditions, the interfacial bond strength of the VPC both without a coupling agent and with KH550 varied with the thermoplastic type in the order PVC > PP > HDPE > LDPE (Figure 2a). The mechanical strength of thermoplastics and wood veneer, and the mechanical interlocking and chemical bonds formed at the interface, affected the interfacial bond strength of the VPC. PP had higher strength than HDPE and LDPE. In addition, PVC is a polar and amorphous polymer, in contrast to the nonpolar PP and HDPE, which provide better interfacial interactions with wood. Thus, the VPC made with PVC had the largest interfacial bond strength under both dry and wet conditions. Previous research showed that low-melt-viscosity polymers could penetrate deeper into the pores and gaps of wood and form better mechanical interlocking, thus giving a high interfacial bond strength [26]. Compared with dry conditions, the VPC under wet conditions had a lower interfacial bond strength (Figure 2c), which may have been caused by poorer wood mechanical properties and the breakage of chemical bonds produced by coupling agents.

Both MDI and KH550 improved the interfacial bond strength of the VPC, consistent with previous studies [27,28]. The strength enhancement was due to the chemical reaction between coupling agents and raw materials, which decreased the content of hydrophilic hydroxyl groups on the wood surface, due to the grafting reaction with the silane of KH550, generating Si-O-C bonds [22]. Urethane bonds were formed by the reaction between the isocyanate groups of MDI and the hydroxyl groups of wood and plastics [24].

The use of MDI as a coupling agent produced a higher interfacial bond strength than KH550. The average interfacial bond strength values for PVC, HDPE, PP, and LDPE under dry conditions were 2.1, 1.7, 1.5, and 1.3 MPa, respectively, and 1.8, 1.7, 1.3, and 1.1 MPa under wet conditions, respectively. Thus, MDI produced a better bonding interface between wood and plastics than KH550. The use of KH550 as a coupling agent weakened the interfacial bond of all VPCs under wet conditions, except for the VPC made with PP (Figure 2b). MDI as a coupling agent greatly strengthened the interfacial bond of the VPC under both dry and wet conditions (Figure 2a,b). The interfacial bond strength of the VPC with KH550 was 43.1–77.6% lower under wet conditions than under dry conditions (Figure 2c), whilst with MDI as the coupling agent, the bond strength was 4.1–18.1% lower under wet conditions than under dry conditions (Figure 2d). Compared with MDI as the only adhesive, the addition of a thermoplastic increased the interfacial bond strength between wood veneers under dry conditions (Figure 2d), indicating the penetration of thermoplastics inside the wood may have increased the mechanical interlocking at the interface. The highest interfacial bond strength of the VPCs was obtained when PVC was used as the thermoplastic, and MDI as the coupling agent, though the VPC made from HDPE with MDI as the coupling agent also had a desirable interfacial bond strength.

### 3.2. Dynamic Mechanical Analysis of VPC

**Figure 3:**
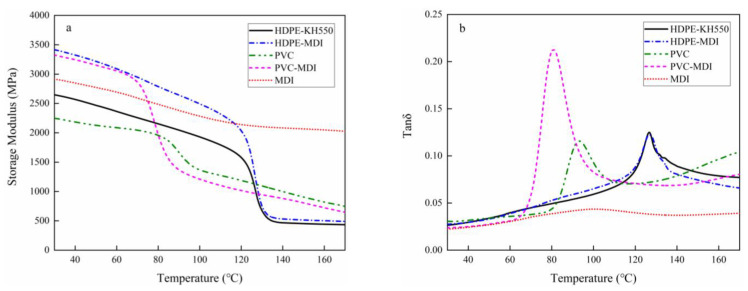
(**a**) Storage modulus and (**b**) loss tangent of VPC with: HDPE as the thermoplastic and KH550 as the coupling agent (HDPE-KH550); HDPE as the thermoplastic and MDI as the coupling agent (HDPE-MDI); PVC as the thermoplastic and no coupling agent (PVC); PVC as the thermoplastic and MDI as the coupling agent (PVC-MDI); and plywood with MDI as the adhesive (MDI).

The normalized storage modulus (*E’*) and loss tangent (*tanδ*) were used to examine the dynamic thermomechanical properties of the VPC, as shown in Figure 3. When the temperature increased, *E’* first gradually decreased for all samples, and then rapidly dropped when the temperature reached softening temperature at 80 °C and 120 °C for PVC and HDPE. Previous studies support these results. For instance, Qi et al. [4] found that the melting temperature range of HDPE is 121.2–151.3 °C, and Li et al. [29] confirmed that PVC exhibited an exothermic peak at 82.8 °C. The VPC could not support a high load when the thermoplastics were completely melted. Both KH550 and MDI greatly increased the storage modulus of the VPC made from PVC and HDPE, indicating that the coupling agents greatly improved the interfacial bond strength, consistent with the results in Figure 2. The interface between the veneer and plastic changed from mechanical interlocking to chemical bonding, which improved the bond between wood and plastics and increased the sample’s rigidity [16]. Figure 3 shows that both MDI and KH550 improved the stiffness of VPC made from PVC and HDPE, with a higher *tanδ*, and MDI displayed better performance than KH550. Figure 3 also shows that PVC as the adhesive increased the plywood’s stiffness compared with using MDI only; however, this advantage became a weakness at temperatures above the PVC softening temperature. Thus, the utilization temperature of wood–thermoplastic composites is limited, but they are suitable for general applications such as furniture, floor, and interior decoration.

### 3.3. Morphological Structure of VPC

**Figure 4:**
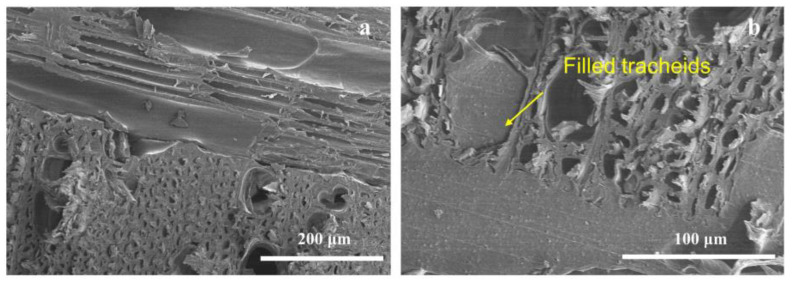
Interfacial SEM micrograph of the VPC made from HDPE: (**a**) without coupling agent; (**b**) with MDI as the coupling agent.

**Figure 5:**
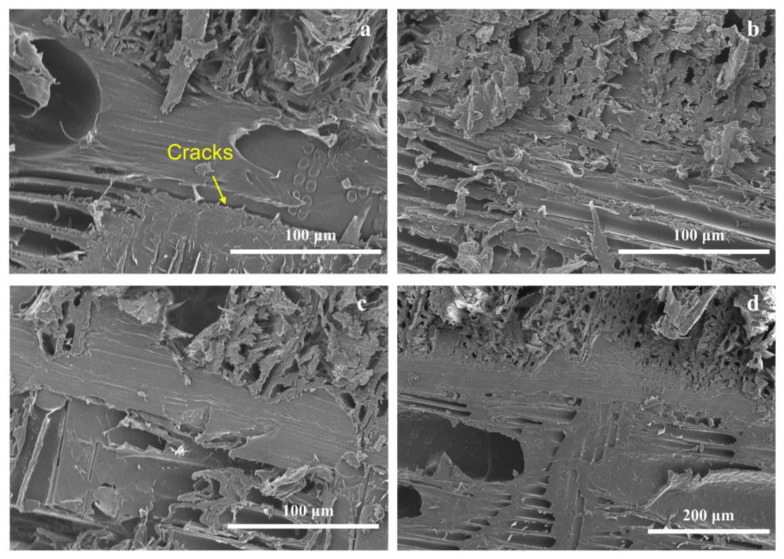
SEM micrograph of the interface between the veneer and: (**a**) LDPE; (**b**) LDPE with KH550 as the coupling agent; (**c**) PVC; (**d**) PVC with KH550 as the coupling agent.

The morphologies of VPCs with different thermoplastic types and coupling agents are shown in Figure 4 and Figure 5. HDPE and PVC entered adjacent tracheids of a larger size, but they barely penetrated the small lumen (Figure 4b and Figure 5d). Cracks were observed between the interfaces of longitudinal and transverse veneers in the VPC without coupling agents (Figure 4a and Figure 5a). On the other hand, VPCs showed a tight interaction, without cracks, when MDI or KH550 was used as the coupling agent (Figure 4b and Figure 5d). Altogether, these results prove that MDI and KH550 improve the interfacial bonds between the wood and thermoplastics, supporting the results for interfacial bond strength shown in Figure 2, and storage modulus in Figure 3.

### 3.4. Mechanical Properties of OSPC

**Figure 6:**
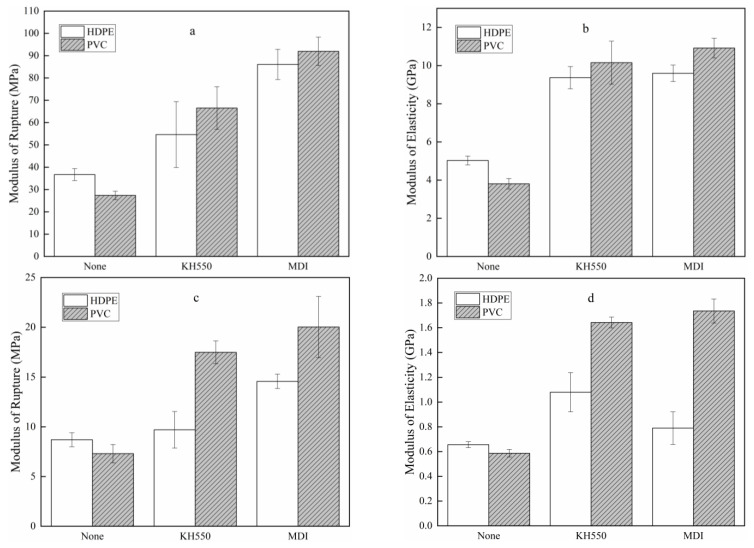
(**a**) MOR in the parallel direction, (**b**) MOE in the parallel direction, (**c**) MOR in the vertical direction, and (**d**) MOE in the vertical direction of OSPC.

Figure 6 shows the modulus of rupture and modulus of elasticity of OSPC. MDI and KH550 improved the MOR and MOE of OSPC in the parallel and vertical directions. MDI performed better than KH550, and the strength showed a greater improvement than the stiffness. The average MOR of OSPC made from PVC or HDPE, with MDI as the coupling agent, was 91.9 MPa and 86.1 MPa in the parallel direction, respectively, which was an increase of 235.9% and 134.5%, respectively, compared with OSPC without a coupling agent. Similarly, their average MOE was 10.9 GPa and 9.6 GPa in the parallel direction, which increased by 186.8% and 90.8%, respectively. A similar trend was obtained for the MOR and the MOE in the vertical direction. 

The OSPC made from PVC without a coupling agent had a lower MOR and MOE in parallel and vertical directions than HDPE-OSPC because PVC is a flexible polymeric matrix with worse mechanical properties than HDPE [30]. However, the mechanical properties improved when MDI and KH550 were applied to strengthen the interface. This indicates that the interfacial bonding between PVC and wood was greatly enhanced by adding MDI and was higher than KH550, corresponding to the findings of Englund et al. [31] and verified by the interfacial bond strengths shown in Figure 2. These results reveal that better interfacial bond strength between wood and thermoplastics improves the mechanical properties of WPC.

### 3.5. OSPC Dimensional Stability 

**Figure 7:**
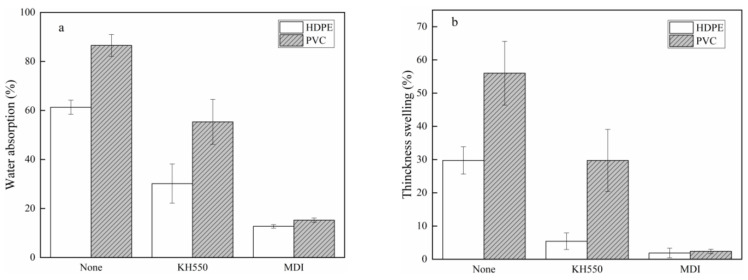
(**a**) Water absorption and (**b**) thickness swelling of OSPC.

Figure 7 shows the effects of the coupling agent and thermoplastic type on the water absorption and thickness swelling after 24 h immersion in water. The average water absorption of the HDPE-OSPC made without coupling agent, with KH550 or MDI was 61.3%, 30.2%, and 12.7%, respectively, and 86.5%, 55.4%, and 15.2% for the PVC-OSPC. The moisture absorption in wood mainly occurs at pores, cracks, and hydrogen-bonding sites [32]. Both KH550 and MDI improved dimensional stability, especially MDI. When MDI was added, the average thickness swelling of the HDPE- and PVC-OSPC decreased by 27.9% and 53.6%, respectively. Coupling agents improved the interfacial bond between wood and plastic by reducing interfacial gaps and water penetration. The PVC-OSPC had a higher water absorption than HDPE-OSPC since PVC is more compatible with poplar water molecules than nonpolar HDPE.

Similarly, the average thickness swelling of the HDPE-OSPC without a coupling agent and with KH550 and MDI as the coupling agent was 29.7%, 5.4%, and 1.9%, respectively, and it was 56.0%, 29.7%, and 2.4%, respectively, for the PVC-OSPC. The addition of KH550 and MDI improved the thickness swelling of the OSPC, and MDI had a larger effect than KH550. The thickness swelling of the OSPC with MDI as the coupling agent was below 2.5%, indicating that these OSPC have potential applications in high-humidity and outdoor environments. The stronger interfacial bonding between wood and thermoplastic produced an OSPC with better dimensional stability for the specific thermoplastic.

## 4. Conclusions

Poplar veneer–thermoplastic composites and oriented strand–thermoplastic composites were fabricated using hot-pressing in this study. It was found that the use of both KH550 and MDI as coupling agents improved the interfacial bond strength between wood and thermoplastics under dry conditions. The use of MDI resulted in a much greater increase in the interfacial bond strength than KH550 under both dry and wet conditions, while KH550 had a negative effect under wet conditions. The better interfacial bond strength between wood and thermoplastics gave OSPC better mechanical properties and dimensional stability. The OSPC fabricated using PVC film and MDI gave the highest mechanical properties, with a MOR of 91.9 MPa and MOE of 10.9 GPa. PVC and MDI are recommended to fabricate WPC with a desirable performance for general use. However, WPC made from PVC was not suitable for high-temperature purposes, while HDPE could withstand higher temperatures. The results will guide the industry to produce high-performance WPC using hot pressing for general applications.

## Figures and Tables

**Table 1:** Experimental design for fabrication of the VPC and OSPC.

Products	Factors	Materials	Coupling Agent Content
VPC	Coupling agent	None	60.0 g/m^2^
		MDI	
		KH550	
	Thermoplastic type	HDPE	
		LDPE	
		PP	
		PVC	
OSPC	Coupling agent	None	2.0 wt%
		MDI	
		KH550	
	Thermoplastic type	HDPE	
		PVC	

## Data Availability

The data presented in this research are available on request from the corresponding author.
